# Cochlear implantation is safe and effective in patients with *MYH9*-related disease

**DOI:** 10.1186/1750-1172-9-100

**Published:** 2014-06-30

**Authors:** Alessandro Pecci, Eva JJ Verver, Nicole Schlegel, Pietro Canzi, Carlos M Boccio, Helen Platokouki, Eike Krause, Marco Benazzo, Vedat Topsakal, Andreas Greinacher

**Affiliations:** 1 Department of Internal Medicine, IRCCS Policlinico San Matteo Foundation and University of Pavia, Piazzale Golgi, 27100 Pavia, Italy; 2 Department of Otorhinolaryngology and Head & Neck Surgery, Rudolf Magnus Institute of Neuroscience, University Medical Center Utrecht, Utrecht, The Netherlands; 3 Service d’Hématologie Biologique and National Reference Center on Inherited Platelet Disorders, Robert-Debré Hospital, Paris, France; 4 Department of Otorhinolaryngology, IRCCS Policlinico San Matteo Foundation and University of Pavia, Pavia, Italy; 5 Department of Otorhinolaryngology, Hospital Italiano de Buenos Aires, Buenos Aires, Argentina; 6 Haemophilia Centre and Haemostasis Unit, Aghia Sophia Children’s Hospital, Athens, Greece; 7 Department of Otorhinolaryngology, Head and Neck Surgery, Ludwig-Maximilian-University, Munich, Germany; 8 Institut für Immunologie und Transfusionsmedizin, Ernst-Moritz-Arndt-Universität, Greifswald, Germany

**Keywords:** *MYH9*-related disease, Genetic deafness, Inherited thrombocytopenia, Cochlear implantation, Fetchner syndrome, Epstein syndrome, May-Hegglin anomaly, Non-muscle myosin

## Abstract

**Background:**

*MYH9*-related disease (*MYH9*-RD) is a rare syndromic disorder deriving from mutations in *MYH9*, the gene for the heavy chain of non-muscle myosin IIA. Patients present with congenital thrombocytopenia and giant platelets and have a variable risk of developing sensorineural deafness, kidney damage, presenile cataract, and liver abnormalities. Almost all *MYH9*-RD patients develop the hearing defect, which, in many individuals, progresses to severe to profound deafness with high impact on quality of life. These patients are potential candidates for cochlear implantation (CI), however, no consistent data are available about the risk to benefit ratio of CI in *MYH9*-RD. The only reported patient who received CI experienced perisurgery complications that have been attributed to concurrent platelet defects and/or MYH9 protein dysfunction.

**Methods:**

By international co-operative study, we report the clinical outcome of 10 patients with *MYH9*-RD and severe to profound deafness who received a CI at 8 institutions.

**Results:**

Nine patients benefited from CI: in particular, eight of them obtained excellent performances with restoration of a practically normal hearing function and verbal communication abilities. One patient had a slightly worse performance that could be explained by the very long duration of severe deafness before CI. Finally, one patient did not significantly benefit from CI. No adverse events attributable to *MYH9*-RD syndrome were observed, in particular no perisurgery bleeding complications due to the platelet defects were seen. Patients’ perioperative management is described and discussed.

**Conclusions:**

CI is safe and effective in most patients with *MYH9*-RD and severe to profound deafness and should be offered to these subjects, possibly as soon as they develop the criteria for candidacy.

## Background

*MYH9*-related disease (*MYH9*-RD) is an autosomal-dominant syndromic disorder deriving from mutations in *MYH9*, the gene for the heavy chain of non-muscle myosin IIA (NMMHC-IIA) [1,2]. *MYH9*-RD is characterized by a complex phenotype. All patients present at birth with thrombocytopenia, platelet macrocytosis, and pathognomonic cytoplasmic inclusions of the mutant protein in leukocytes; most of them subsequently develop sensorineural hearing loss, proteinuric nephropathy, presenile cataract, and/or alterations of liver enzymes [3,4]. *MYH9*-RD encompasses four syndromes that have been considered for many years as distinct disorders, May-Hegglin Anomaly (MHA, MIM 155100), Sebastian syndrome (SBS, MIM 605249), Fechtner syndrome (FTNS, MIM 153640), and Epstein syndrome (EPTS, MIM 153650). The identification of *MYH9* as the gene responsible for all of these syndromes led to demonstration that MHA, SBS, FTNS, and EPTS actually represented different clinical presentations of the same condition, for which the definition of *MYH9*-RD has been introduced [5-7].

Sensorineural hearing loss is the most frequent non-congenital manifestation of the *MYH9*-RD. A recent analysis of 255 patients showed that deafness was present in about one-half of cases at a mean age at evaluation of 35 years, and was expected to develop over time in almost all cases [8]. The severity of hearing impairment is variable among different patients: while in some *MYH9*-RD subjects the hearing defect is mild or moderate even at advanced age, in other patients hearing loss presents during childhood and progresses to profound deafness within the first decades of life [8-10]. Thus, in many *MYH9*-RD patients deafness greatly contributes to patients’ disability.

Non-muscle myosin-IIA is a cytoplasmic myosin expressed in most cell types and tissues, including the inner ear [11-14]. As all conventional myosins, it has a hexameric structure formed by one heavy chain (NMMHC-IIA) dimer and two pairs of light chains. Each NMMHC-IIA molecule recognizes an N-terminal head domain (HD) responsible for enzymatic activity and a C-terminal tail domain (TD) mainly responsible for myosin assembly [15]. Genotype-phenotype studies showed that patients with mutations in the HD have a higher risk of early-onset and severe deafness than subjects with mutations in the TD [8-10]. Of note, the *MYH9* gene was identified as responsible also for a non-syndromic form of autosomal-dominant deafness, designated DFNA17 (MIM 603622), in two pedigrees carrying the same HD mutation, p.R705H [12,16].

Cochlear implantation (CI) is a potential option for patients with *MYH9*-RD and severe to profound deafness, however, no consistent data are available about the risk to benefit ratio of CI in this condition. Reduced platelet counts of *MYH9*-RD patients obviously result in an increased risk of bleeding complications during of after surgery, and decision to perform CI and peri-operative management requires the co-operation of the hematologist with the ENT surgeon. To date, only one patient with a clinical diagnosis of EPTS who had received CI has been reported [17]. This patient benefited from CI, but surgery was complicated by delayed wound healing and subsequent severe chronic infection, which were attributed to the chronic thrombocytopenia and defective tissue repair due to the impaired NMMHC-IIA function [17]. The efficacy of CI in *MYH9*-related deafness is made even more uncertain in view of the discordant results obtained in non-syndromic deafness DFNA17: members of both the reported DNFA17 pedigrees received CIs, with good outcomes in one family and very poor results in the other one [16,18].

Here we report the clinical outcome of CI in 10 patients with *MYH9*-RD and severe to profound deafness. Our results provide evidence that CI should be offered to patients affected by this condition.

## Patients and methods

This study includes 10 *MYH9*-RD patients who received CI between 1987 and 2009 at 8 different ENT centres in The Netherlands (1 centre/3 patients), Italy (3 centres/3 patients), France, Germany, Greece, and Argentina (1 patient each) [19-22]. Mutational screening of the *MYH9* gene was performed at 3 different institutions by previously reported methods [19,20]. Immunofluorescence assay for the identification of NMMHC-IIA leukocyte inclusions was carried out as previously described [19,23]. Severity of bleeding was graded according to the WHO bleeding score, except for severity of intraoperative bleeding during CI surgery, which was described according to Boezaart et al. [24]. Pure tone audiometric examinations were performed at the different ENT centers by standard methods. Pure tone average (PTA) was calculated using air conduction thresholds at 500, 1000, 2000 and 4000 Hz. Speech discrimination tests were administered at the different ENT centers too. Despite some differences in the utilized tests, all of them included the assessment of the percentages of discrimination of words and sentences at a conversation voice from an open list, which were therefore used to describe the CI outcomes. Unless otherwise specified, the speech perception scores are mentioned without the use of visual support. To describe short-term outcome of CI, evaluation at 6 or 12 months after switch-on of the implanted device was reported; for long-term outcome, evaluation at the last follow-up visit was used. The investigation was approved by the Institutional Review Board of the IRCCS Policlinico San Matteo Foundation, Pavia, Italy. All the patients or their legal guardians gave written informed consent for this retrospective study, which was conducted according to the declaration of Helsinki.

## Results

### Patients

The main clinical and laboratory features of the 10 enrolled patients are summarized in Table 1. In all the cases, diagnosis of *MYH9*-RD based on the findings of congenital thrombocytopenia, giant platelets, and identification of pathognomonic NMMHC-IIA leukocyte inclusions by immunofluorescence assay [19], and was confirmed by identification of the causative *MYH9* mutation. In 8 cases the mutations affected the HD of NMMHC-IIA and in two cases the TD. Finally, for 7 patients the *MYH9*-RD was transmitted in an autosomal-dominant manner, while three patients had sporadic forms deriving from *de novo* mutations (Table 1). Table 2 reports the management of bleeding risk on the occasions of previous surgery and respective outcomes.

**Table 1:** **Essential clinical features of 10 *MYH9*-RD patients who received cochlear implant**

**Patient/family**	**Age**^**1**^**/gender**	**Inheritance**	**NMMHC-IIA mutation (domain)**^**2**^	**Leukocyte inclusions, MGG/IF**	**Platelet count ×10**^**9**^**/L, automated/** **microscopic**	**Spontaneous bleeding WHO**^**3 **^**(type of bleeding)**	**Kidney involvement**	**Cataract**	**Other relevant information**	**Ref.**
1/1	34/F	Sporadic	p.R702C (HD)	Yes/Yes	8/14	2 (easy bruising, menorrhagia)	Kidney transplantation	No	Chronic immunosuppressive drugs administration^4^	A
2/2	40/M	Sporadic	p.R702C (HD)	No/Yes	24/31	1 (easy bruising)	Nephrotic range proteinuria, CRF	No	Previous splenectomy; history of recurrent otitis media (bilateral); HCV hepatitis	A
3/3	43/M	Sporadic	p.R702S (HD)	No/Yes	21/25	2 (easy bruising, epistaxis)	Proteinuria	No	Previous splenectomy; history of chronic otitis media (bilateral)	A
4/4	72/M	AD	p.A95D (HD)	Yes/Yes	70/nd	0	No	No	History of chronic otitis media (left ear) with TM perforation	A
5/5	27/F	AD	p.D1424Y (TD)	Yes/Yes	10/80	0	No	No	None	A
6/6	50/F	AD	p.W33R (HD)	Yes/Yes	19/nd	1 (easy bruising)	No	No	None	B
7/7	12/M	AD	p.R705H (HD)	Yes/Yes	96/nd	0	No	No	None	C
8/7	30/F	AD	p.R705H (HD)	Yes/Yes	115/nd	1 (easy bruising)	No	No	None	C
9/7	46/F	AD	p.R705H (HD)	Yes/Yes	142/nd	1 (easy bruising)	No	No	None	C
10/8	54/F	AD	p.D1424N (TD)	Yes/Yes	39/nd	2 (easy bruising, menorrhagia)	No	No	None	D

Notes: ^**1**^ = age at time of cochlear implant. ^**2**^ **=** NMMHC-IIA domain affected by mutation. ^**3**^ = according to WHO bleeding scale: grade 0, no bleeding; grade 1, only cutaneous bleeding; grade 2, mild blood loss; grade 3, gross blood loss, requiring transfusion; grade 4, debilitating blood loss, retinal or cerebral associated with fatality. ^**4**^ = immunosuppressive treatments (tacrolimus, mycophenolate mofetil, and steroids) for previous kidney transplantation.

*Abbreviations:NMMHC-IIA* non-muscle myosin heavy chain IIA, *HD* head domain, *TD* tail domain, *ref*. reference, for previously reported patients, *AD* autosomal-dominant, *CRF* chronic renal failure, *TM* tympanic membrane, *nd* not determined.

References: A = Pecci et al., [8]. B = Saposnik et al., [20]. C = Verver et al., [21] D = Greinacher et al. [22].

**Table 2:** **Surgical history of 10 *MYH9*-RD patients who received cochlear implant**

**Patient/family**	**Platelet count ×10**^**9**^**/L, automated/microscopic**	**Type of surgery**	**Prophylaxis for bleeding**	**Perioperative bleeding (WHO grade)**
1/1	8/14	Kidney transplantations (twice), nephrectomy, laparotomy (peritonitis)	Platelet transfusions before surgery, on all interventions	None
2/2	24/31	Splenectomy	Platelet transfusions	None
3/3	21/25	Tonsillectomy, splenectomy, removal of pilonidal cyst	Platelet transfusions before surgery, on all interventions except tonsillectomy	Bleeding after tonsillectomy (2)
4/4	70/nd	None	-	-
5/5	10/75	None	-	-
6/6	19/nd	Amygdalectomy, removal of tympanic glomus tumor, reduction of calcaneous fracture, ankle arthrodesis, amygdalectomy	Platelet transfusions before surgery, on all interventions	None
7/7	96/nd	Positioning of ventilation tubes	None	None
8/7	115/nd	Appendectomy, ovariectomy, laparoscopic removal of adhesions, episiotomy	None	Bleeding after episiotomy (2)
9/7	142/nd	Fixation of ankle fracture	None	None
10/8	45/nd	Adenoidectomy, nasal septoplasty, hysterectomy	Tranexamic acid before and after surgery, on all interventions	None

*Abbreviations*: *nd* = not determined.

### Sensorineural deafness

**Figure 1:**
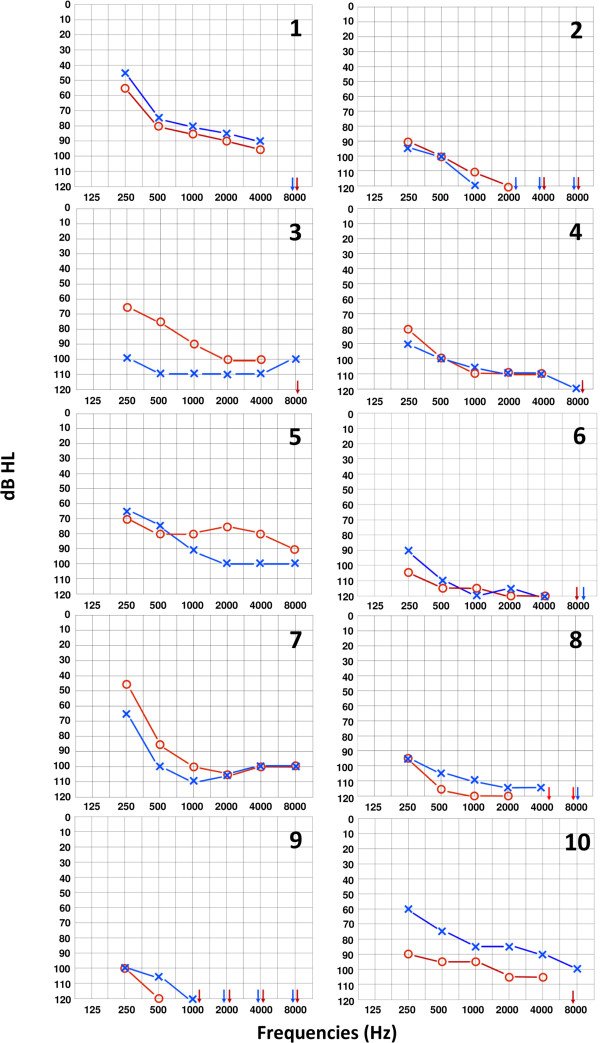
**Audiometric tracings prior to CI in 10 patients with *MYH9*-RD.** Each audiogram reports the air conduction hearing thresholds of the analyzed patients. Patients are identified by numbers on top right, which correspond to identification numbers reported in Tables. Hearing threshold is defined as the minimum sound intensity (presented at different frequencies) that can be still perceived by the individual during the audiometric examination. Hearing thresholds were measured in decibel hearing level (dB HL) and frequencies ranged from 0.25 to 8 kHz. The right ear is indicated by the red symbol “O” and the left ear by the blue symbol “X”. Severe deafness is defined by mean hearing thresholds worse than 70 dB HL, profound deafness by mean hearing thresholds worse than 90 dB HL. Arrows indicate hearing thresholds worse than 120 dB HL.

All the patients had bilateral sensorineural hearing loss that at diagnosis affected only or predominantly the high tones and subsequently progressed toward severe to profound deafness involving all frequencies. Mean age at onset was 16.6 years and speech development was normal in all the cases. Data about progression of deafness and the findings at the last available audiometric examination before CI are reported in Table 3 and Figure 1. Mean duration of deafness before CI was 24.2 years (range, 7–55), and mean duration of severe deafness (PTA >70 dB NHL) before CI was 8.0 years (range, 2–22). In all the subjects CI was performed because of poor benefit deriving from conventional hearing aids: all the patients had an open list speech recognition score lower than 50% in the best aided conditions without lip reading.

**Table 3:** **Age at onset, progression, and severity of sensorineural deafness before CI in 10 *MYH9*-RD patients**

**Patient/ family**	**Age at onset**^**1**^	**Age at first use of hearing aids**	**Age at evolution toward PTA > 70 dB NHL**	**Age at CI**	**PTA before CI right/left**^**2**^
1/1	20	23	24	34	87/82
2/2	20	30	32	40	115/120
3/3	8	34	34	43	91/110
4/4	17	64	70	72	107/106
5/5	20	25	25	27	78/91
6/6	25	48	48	50	118/116
7/7	3	5	8	12	97/104
8/7	4	13	19	30	121/111
9/7	19	21	24	46	126/121
10/8	30	33	44	54	100/85
**Mean (SD)**	16.6 (9)	29.6 (17)	32.8 (17)	40.8 (16)	

Notes: ^**1**^ = self-reported. ^**2**^ = the respective audiometric tracings are showed in Figure 1.

### CI surgery

Nine patients received unilateral CI, whereas patient 5 received a sequential bilateral implantation at the ages of 27 (left) and 28 (right). In all patients preoperative CT scan and/or MRI showed no anomalies of the temporal bone at the implanted side. Table 4 reports prophylaxis for bleeding complications and perioperative bleeding in each subject. All patients received prophylactic intravenous antibiotics for 1 to 10 days (median, 3 days) and no infectious episodes were reported. Perisurgery adverse events occurred in two patients. In patient 8, a small hematoma was found at the site of the surgical wound. One stitch was then removed and by giving pressure the hematoma was drained without any further complications. In patient 9, cochleostomy was complicated by massive gusher that was treated by placing a lumbal drain and application of gelfoam at the cochleostomy. Nevertheless, all the electrodes could be correctly introduced. Moreover, postoperatively she developed an eardrum perforation for which a myringoplasty was successfully performed. Finally, normal wound healing was reported for all the patients.

**Table 4:** **CI surgery in 10 patients with *MYH9*-RD**

**Patient/family**	**CI side**	**Surgical approach**^**1**^	**Device**	**Prophylaxis for bleeding**	**Intraoperative bleeding**^**2**^	**Postoperative bleeding**
1/1	R	Round window	Digisonic SP (Neurelec)	Platelet apheresis, 1U pre + 1U post	1	None
2/2	R	Round window	Nucleus CI24RE (Cochlear)	Platelet apheresis, 1U pre	3	None
3/3	L	Round window	Maestro Concerto Flex 28 (Med-el)	Platelet apheresis, 1U pre + 1U post	1	None
4/4	R	Round window	Nucleus Freedom CI24RE (Cochlear)	None	1	None
5/5	L + R	Cochleostomy	Nucleus Mini22 (Cochlear)	nd	1	None
6/6	L	Round window	Digisonic SP (Neurelec)	Platelet apheresis, 1U pre phenocompatible	1	None
7/7	L	Cochleostomy	Nucleus CI512 (Cochlear)	None	1	None
8/7	R	Cochleostomy	Nucleus CI24R (CA) (Cochlear)	None	2	Hematoma at the site of surgical wound
9/7	L	Cochleostomy	Mini-system 22 CI22M (Cochlear)	None	1	None
10/8	R	Round window	Maestro Concerto Flex 28 (Med-el)	Tranexamic acid 1 g i.v. pre, then 1500 mg/day p.o. post for 7 days	1	None

Notes: ^**1**^ = use of cochleostomy or round window for positioning of the device. ^**2**^ = Bleeding score adapted according to Boezaart et al., [24]: Grade 1: minimal suction. Grade 2: slight bleeding, infrequent suction. Grade 3: brisk bleeding, frequent suction. Grade 4: strong bleeding, bleeding covers surgical field after removal of suction before instrument can perform manoeuver; Grade 5: uncontrolled bleeding, bleeding out of mastoid cavity on removal of suction. *Abbreviations*: *pre* = preoperative, *post* = postoperatively.

### Outcome of CI

Mean follow-up after CI was 9.3 years (range: 1–25). Table 5 summarizes the results of speech perception tests administered 6 or 12 months after the switch-on of the device and at the last follow-up evaluation. The overall clinical outcome of the CI in the 10 patients can be summarized as follows. (i) Eight patients obtained excellent short-term results, with restoration of a practically regular hearing function and speech perception since the evaluation at 6–12 months. For seven of them, this performance was maintained unchanged until the last follow-up examination carried out at 1 to 9 years after CI (mean, 4.6). For patient 5, such an excellent performance was maintained for about 10 years. Subsequently, she experienced a progressive deterioration of her hearing ability and speech perception. Unfortunately, the specific reasons for this deterioration of CI performance could not be clarified, as the patient refused any further investigations. (ii) Patient 9 obtained worse short-term results with respect to the majority of other responders (Table 5). However, her ability in speech discrimination progressively increased over time, until reaching a maximum sentence score without visual support of 94% at 13 years follow-up (contextual word score 50%), and a maximum word score of 64% at 17 years follow-up (sentence score 85%). However, about 13 years after CI, she developed a progressive cognitive defect due to Alzheimer disease that probably affected the results of speech perception tests and the standard follow-up had to be stopped for severe dementia 19 years after CI. (iii) Patient 6 experienced no significant short- or long-term improvement of hearing ability after CI. On the last evaluation, this patient was able to discriminate 66% and 85% of words (open and closed list, respectively) and 100% of sentences by lip reading and CI, that was a better performance than by lip reading alone (data not shown), suggesting that CI brought some additional information to her. However, her recognition score was 0% for both words and sentences by using the CI alone. The subsequent examinations did not identify a definite cause for this poor outcome.

**Table 5:** **Results of speech discrimination tests administered 6 or 12 months after the switch-on of the device (short-term outcome) and at the last follow-up evaluation (long-term outcome) in 10 *MYH9*-RD patients**

**Patient/family**	**Short-term outcome**		**Long-term outcome**		
	**Discrimination of words (-HA)**^**1 **^**[%]**	**Discrimination of sentences (-HA)**^**1 **^**[%]**	**Time after CI (years)**	**Discrimination of words (-HA)**^**1 **^**[%]**	**Discrimination of sentences (-HA)**^**1 **^**[%]**
1/1	90 (88)	95 (95)	5	100 (95)	100 (100)
2/2	90 (nd)	90 (nd)	4.5	92 (nd)	90 (nd)
3/3	95 (90)	95 (95)	4.5	98 (97)	100 (100)
4/4	80 (nd)	100 (nd)	5	90 (nd)	100 (nd)
5/5	90 (nd)	98 (nd)	25	nd	nd
6/6	0 (0)	0 (0)	17	0 (0)	0 (0)
7/7	97 (88)	100 (nd)	3	100 (85)	100 (nd)
8/7	90 (75)	99 (nd)	9	nd (85)^**2**^	nd (100)^**2**^
9/7	4 (nd)	24 (nd)	19	nd (42)^**2**^	nd (65)^**2**^
10/8	95 (nd)	82 (nd)	1	100 (nd)	82 (nd)

Notes: ^**1**^ = whenever recorded, results obtained without the contralateral external hearing aid (HA) are reported in parentheses (-HA). ^**2**^ = At the time of their last follow-up evaluations, patients 8 and 9 did not usually utilize a hearing aid: therefore, they were tested with CI alone. *Abbreviations*: *CI* = cochlear implant, *nd* = not determined.

The speech perception scores are intended without the use of visual support.

## Discussion

Patients affected by *MYH9*-RD with severe to profound deafness are potential candidates for CI. To date the only information about outcome of this procedure in *MYH9*-RD derives from a single case report. This subject benefited from CI, but surgery was complicated by delayed wound healing attributed to the chronic thrombocytopenia and/or NMMHC-IIA dysfunction. Moreover, the effectiveness of CI in *MYH9*-related deafness was questioned in view of the discordant results obtained in two families with the non-syndromic deafness DFNA17 [25]. In order to provide consistent information on the risk to benefit ratio of CI in *MYH9*-RD, we have gathered the data of 10 patients who received CI at 8 different institutions.

CI was effective in improving the hearing ability in 9 out of 10 *MYH9*-RD patients, while one subject did not take advantage from the procedure. In particular, 8 responders obtained excellent performances, with restoration of a practically regular hearing function since evaluation at 6–12 months after the switch-on of the implant. All of them referred restoration of the ability to engage in a normal conversation even in a noisy environment; they also reported good performance with phone conversations or listening to devices such as radio or television. These excellent responders were characterized by durations of severe deafness prior to CI ranging from 2 up to 11 years (mean, 7.0 years). Moreover, CI performance was similarly good in patients with different total durations of deafness before CI (7 to 55 years) and with different ages at implantation (childhood up to 72 years). Finally, similarly good performances have been obtained in 6 patients with mutations hitting the HD of NMMHC-IIA or in 2 patients with mutations in the TD, suggesting that CI outcome was independent on the specific *MYH9* alteration. One subject (patient 9) benefited from CI, but performance was not as good as in the other responders. In fact, results of her speech perception tests were poor at the short-term evaluation after switch-on; however, her speech discrimination scores ameliorated over time until reaching fairly good levels that were maintained until 17 years after implantation. This patient differed from the other ones for the markedly longer duration of severe deafness before CI, i.e. 22 years. In CI responders affected by other forms of post lingual deafness, duration of severe deafness prior to implantation was a major predictor of CI performance [26,27]. We therefore hypothesize that this feature affected the CI performance also in this subject and we suggest that CI should be offered to *MYH9*-RD patients shortly after they develop criteria for candidacy.

On the other hand, the reasons why the patient 6 did not benefit from CI remain undetermined. Among the different factors that could potentially affect CI outcome [26,28,29], we could not identify any feature of this patient explaining her poor response. The patient carried the p.R33W mutation of the HD of NMMHC-IIA, which represents a rare variant described in only one other *MYH9*-RD patient [30]. However, the clinical pictures of both patients with p.W33R do not suggest that this mutation induces a particularly severe NMMHC-IIA dysfunction with respect to other HD mutations.

CI surgery was carried out without major bleeding complications. In 4 cases with severe thrombocytopenia, bleeding risk was managed by prophylactic transfusion of 1–2 apheresis platelet concentrates. One center with experience in the care of *MYH9*-RD patients routinely uses tranexamic acid to prepare for surgery patients with moderate thrombocytopenia [31], and successfully used this drug for prophylaxis of patient 10. In 4 patients with automated platelet counts ranging from 70 to 129 × 10^9^/L prophylaxis for bleeding was not deemed necessary. On the whole, intraoperative bleeding was minimal in 8 patients and moderate in two cases; no postoperative bleedings occurred, with the exception of the formation, in one patient, of a hematoma at the site of surgical wound, which was drained by removing one stitch without any clinically relevant consequences. In clinical practice, two aspects should be considered for management of perioperative bleeding risk of *MYH9*-RD patients. First, routine automated cell counters usually underestimate platelet counts of these patients. In fact, electronic instruments identify platelets mainly based on their size and fail to recognize very large platelets typical of *MYH9*-RD [32]. Thus, microscopic counting should be used to assess the actual platelet counts of *MYH9*-RD patients for their proper management. Secondly, since *in vitro* platelet function in *MYH9*-RD is normal or only slightly reduced, the indication for prophylactic transfusions can be reasonably based on the general recommendations for thrombocytopenias. Recent guidelines recommend prophylaxis for patients with platelet counts below 100 × 10^9^/L before surgery at critical sites, and this threshold should be considered for CI [33]. On another line, none of the patients experienced complications related to delayed wound healing. It is therefore unlike that the complications observed in the previously reported *MYH9*-RD patient who received CI were dependent on factors specific to the disease [17]. Finally, three of our patients had conditions leading to increased risk of infection that are rather frequent among *MYH9*-RD patients [3]: two patients had been splenectomized because of a previous misdiagnosis with immune thrombocytopenia and one patient was on immunosuppressive treatment after kidney transplantation. None of them experienced infectious complications after administration of standard antimicrobial prophylaxis. We therefore conclude that CI is a safe procedure in *MYH9*-RD patients whenever adequate prophylactic interventions are carried out.

Pathogenesis of *MYH9*-related deafness is still unclear. Studies on mouse inner ear showed that NMMHC-IIA is extensively localized in the hair cells of the organ of Corti, the spiral ligament and the spiral limbus, with only minimal expression within the spiral ganglion [12,13]. In hair cells, NMMHC-IIA is abundantly expressed in stereocilia [14]. Given that CI bypasses hair cells by directly stimulating the spiral ganglion, the finding that most *MYH9*-RD patients have excellent CI performances is consistent with the NMMHC-IIA expression pattern observed in animals and with the conclusion that *MYH9* mutations primarily damage the hair cells. Altogether, these observations strengthen the notion that CI outcome is better in patients with deafness caused by defects of genes primarily expressed in the hair cells/membranous labyrinth as opposed to mutations causing spiral ganglion pathology [25,34], and further point out the importance of genetic testing in CI candidates. The introduction of massively parallel sequencing technology led to recent development of approaches for an efficient screening of all known deafness genes simultaneously [35-37]. The identification of definite correlations between genotype and CI outcome will pave the road to a tailored patients’ management and reduce the likelihood of ineffective CIs and unnecessary costs in healthcare.

## Conclusions

This study provides evidence that CI is safe and highly effective in restoring hearing ability in most patients with *MYH9*-RD and severe to profound deafness. This procedure should therefore be offered to these subjects, possibly as soon as they develop the criteria for candidacy.

## Abbreviations

*MYH9*-RD: *MYH9*-related disease; NMMHC-IIA: Non-muscle myosin heavy chain IIA; CI: Cochlear implantation.

## Competing interests

The authors declare that they have no competing interests.

## Authors’ contibutions

AP designed the research, acquired data, analyzed and interpreted data, and drafted the manuscript. EJJV, NS, PC, CMB, HP, EK, MB, and VT acquired data, analyzed and interpreted data, and critically revised the manuscript. AG designed research, analyzed and interpreted data, and drafted the manuscript. All the authors revised and accepted the final version of the manuscript.
